# Enhanced Drug Loading Capacity Using the Dual Metformine–Dexketoprofren Salt on Nanoapatite Materials

**DOI:** 10.1021/acs.molpharmaceut.5c00264

**Published:** 2025-04-25

**Authors:** Francisco J. Acebedo-Martínez, Alicia Domínguez-Martín, Carolina Alarcón-Payer, Cristóbal Verdugo-Escamilla, Jaime Gómez-Morales, Duane Choquesillo-Lazarte

**Affiliations:** † Laboratorio de Estudios Cristalográficos, 16379 IACT-CSIC, Avda. de las Palmeras 4, 18100 Armilla, Spain; ‡ Department of Inorganic Chemistry, Faculty of Pharmacy, 16741 University of Granada, 18071 Granada, Spain; § Servicio de Farmacia, Hospital Universitario Virgen de las Nieves, 18014 Granada, Spain

**Keywords:** nanoapatite, metformin, dexketoprofen, multicomponent pharmaceutical materials, drug delivery

## Abstract

Both apatite nanoparticles and multicomponent pharmaceutical materials have proved the ability to significantly improve the bioavailability of different drugs using different strategies. Herein, the use of nanoapatite is proposed as a promising vehicle for advanced drug delivery of multicomponent pharmaceutical materials. To this purpose, the full synthesis and comprehensive characterization of apatite nanoparticles and the molecular pharmaceutical salt metformin–dexketoprofen are reported, paying special attention to the improvements regarding solubility and stability of the novel materials compared to the parent active pharmaceutical ingredients, as well as the drug loading capacity enhancement achieved in nanoapatites. Our results evidence the potential of the presented novel strategy, enhancing the dexketoprofen-loading a remarkable 50-fold when compared to native drug, thanks to the improvement of solubility achieved via salt-formation (567 and 168 mg/mL at pH 6.8 and 1.2, respectively), thus expecting improved therapeutic outcomes.

## Graphical abstract

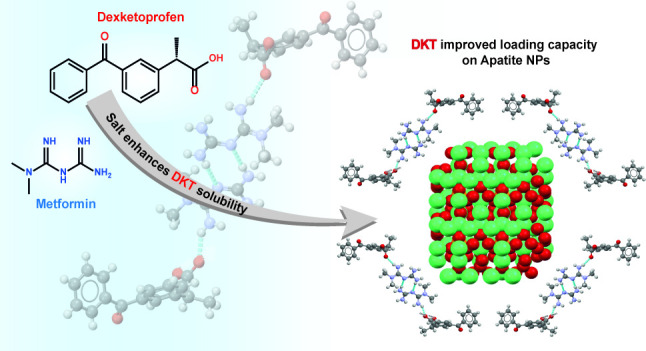

## 1 Introduction

Apatite (Ap), the main inorganic component of bones and teeth, is defined as a non-stoichiometric calcium phosphate, which exhibits deficiencies in Ca^2+^ and OH^–^ ions and ionic substitutions of CO_3_^2–^, Na^2+^, Mg^2+^, as well as other minor elements.^1^ Interestingly, citrate molecules strongly absorbed on an Ap surface.^2^ The numerous ionic groups on the surface, along with a nanometric size, give Ap a higher solubility than its stoichiometric counterpart hydroxyapatite [Ca_5_(OH)­(PO_4_)_3_], the most stable and insoluble phase of calcium phosphates.^3,4^ This difference of solubility, combined with the presence of a non-apatitic hydrated layer, is crucial for ionic adsorption and exchange, allowing the interaction with organic molecules and the applicability of Ap as a drug delivery system.^5,6^

Several studies have demonstrated the potential of nanotechnology-based drug delivery systems to enhance drug solubility and bioavailability, showing promising results in controlled drug release and increased therapeutic efficacy, particularly for hydrophilic drugs.^7–10^ In this area, Ap nanoparticles (nAp) have shown excellent potential for enhancing the bioavailability, adsorption, and distribution of different drugs and minimizing side effects and toxicity associated with accumulation phenomena.^1,11–13^ Moreover, their nanosize provides an extensive surface area, which enables the adsorption of high amounts of active molecules.^14,15^ However, the solubility of the active molecule is a limiting factor that directly influences the amount of material that can be effectively incorporated, as the achievable concentration of the drug in solution is minimal compared to the loading capacity of the nAp. This issue makes it particularly difficult to combine nAp with drugs from class II and IV of the biopharmaceutical classification system (BCS), known for their low solubility in aqueous media.^16,17^

One interesting strategy to improve the solubility of poorly soluble active pharmaceutical ingredients (APIs) that has gained a lot of interest in the last decades is the development of multicomponent pharmaceutical materials (MPMs).^18–23^ MPMs are crystalline materials formed by at least one API and another molecule that is incorporated in the crystal structure, known as a coformer, which must not be toxic or present adverse side effects. The new structure formed between the API and the coformer is maintained through non-covalent interactions, allowing the design of new crystalline materials with unique properties while maintaining the innate activity of the drug.^24–26^

In previous works, our team has demonstrated the potential of metformin (MTF) to effectively form pharmaceutical salts and modulate the physicochemical properties of acidic drugs, thanks to its strong basic nature.^27,28^ MTF is a biguanide antihyperglycemic agent used as a first-line pharmacological treatment in the management of type 2 diabetes due to its efficacy, safety profile, and low costs for patients.^29^ On the other hand, NSAIDs are widely used to reduce pain and inflammation and bring down fever, thus being one of the most commonly prescribed medications worldwide.^30,31^ It is worth mentioning that the combined use of NSAIDs and Ap for bone repair procedures is a common practice, as sustained inflammatory processes tend to inhibit the bone repair process.^32–35^

This work aims to present an innovative approach to overcoming the solubility-associated problems in drug loading operations. For this purpose, a new molecular salt of MTF and dexketoprofen (DKT) (Figure 1) will be synthesized and used for drug-loading operations using biomimetic nAp. In this strategy, the novel salt will increase the solubility of the anti-inflammatory drug in aqueous solutions, addressing the solubility challenges that typically limit the use of drug delivery systems with poorly soluble active molecules.

**Figure 1:**
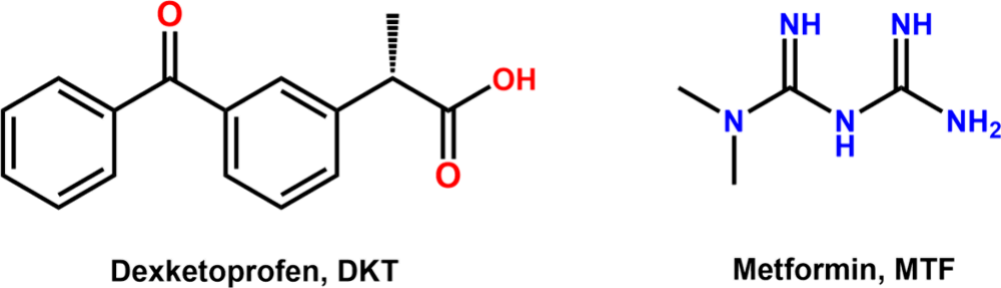
Chemical formula of dexketoprofen (DKT) and metformin (MTF) drugs.

## 2 Experimental Section

### 2.1 Synthesis of nAp

nAp was obtained by thermal decomplexation of Ca^2+^/citrate/phosphate/carbonate solutions.^36^ In this procedure, 50 mL of Na_2_HPO_4_ (0.06 M) and Na_2_CO_3_ (0.1 M) were combined with 50 mL of CaCl_2_ (0.1 M) and Na_3_(cit) (0.2 M), working at 4 °C. The pH of the resulting mixture was adjusted to 8.5 using diluted HCl. Immediately, the solution was transferred to a water bath at 80 °C. After 4 h, the nAp suspension was placed in an oven at 80 °C for 96 h, allowing enough time for Ap maturation. Finally, nAp was recovered through several cycles of centrifugation and washing with Milli-Q water (6 cycles at 9,000 rpm for 9 min each). The resulting precipitate was lyophilized for 12 h (−50 °C and −3 mbar) to obtain a homogeneous powder, which was stored at room temperature until use.

### 2.2 Synthesis of MTF–DKT

#### 2.2.1 Neutralization of MTF–HCl

The MTF-free form is not commercially available due to its low stability and high reactivity. Therefore, a neutralization process of MTF·HCl is necessary. Ten mmol of MTF·HCl (1.656 g) and 10 mmol of NaOH (0.4 g) were stirred in 60 mL of 2-propanol at room temperature in a sealed glass beaker to prevent evaporation. After 24 h, the solution was filtered using 0.22 μm syringe filters to remove the NaCl (insoluble in isopropanol). The filtrate (containing MTF soluble in 2-propanol) was transferred to a vacuum-assisted rotary evaporator set to 40 °C and 30 rpm. Once the solvent was removed, MTF precipitate was collected and stored in light-opaque, sealed tubes until use. The purity of MTF was confirmed repeatedly before each use via powder X-ray diffraction (PXRD) (Figure S1, Supporting Information).

#### 2.2.2 Mechanochemical Synthesis

The mechanochemical synthesis of MTF–DKT was carried out via liquid-assisted grinding (LAG) in a Retsch MM2000 ball mill operating at a frequency of 25 Hz and room temperature. In these operations, a 1:1 mixture of MTF (0.5 mmol, 64.58 mg) and DKT (0.5 mmol, 127.145 mg), 100 μL of ethanol, and two stainless steel balls of 7 mm diameter were placed in stainless steel jars and shaken for 30 min. All products obtained were analyzed by PXRD to determine the formation of a new MPM, its crystallinity, and purity. All operations were repeated in triplicate to ensure reproducibility.

#### 2.2.3 Preparation of Single Crystals

Single crystals of MTF–DKT were obtained through slow evaporation (1 day) at room temperature of saturated solutions of the LAG product by using ethanol. Suitable crystals for single-crystal X-ray diffraction (SCXRD) analysis and structure determination were carefully separated from the solution.

### 2.3 nAp Loading with MTF–DKT

The nAp functionalized with MTF–DKT were obtained through chemical deposition, following the methodology described by Carmona et al.^37^ In these experiments, nAp were mixed with an aqueous solution of MTF–DKT, resulting in an aqueous suspension of nAp. Specifically, 50 mg of nanoparticles was combined with 1 mL of solution, with increasing concentrations of MTF–DKT. The concentrations used for loading the nAp were determined after assessing the solubility of the salt. The same methodology was followed using solutions of DKT to compare the loading capacity of the salt with the original NSAID. After 4 h of stirring, the suspension was lyophilized for 24 h (−50 °C and −3 mbar), and the solid precipitate was stored at room temperature until characterization.

### 2.4 Characterization Techniques

#### 2.4.1 X-ray Diffraction Analysis

PXRD analysis was performed at room temperature on a Bruker D8 Advance Vαrio diffractometer (Bruker-AXS, Karlsruhe, Germany) equipped with a LYNXEYE detector and Cu Kα1 radiation (1.5406 Å). The angular range and sampling time depend on the samples analyzed. For inorganic Ap samples, the diffractograms were recorded over an angular range of 5–70° (2θ) with a step size of 0.02° (2θ) and a total measurement time of 4 h. For organic samples, the angular range was 5–50° (2θ) with a step size of 0.02° (2θ) and a total measurement time of 30 min.

SCXRD data were acquired at room temperature on a Bruker D8 Venture diffractometer (Bruker-AXS, Karlsruhe, Germany) using Cu Kα radiation (λ = 1.54178 Å). The data were processed with the APEX4 suite.^38^ The structure was solved with intrinsic phasing^39^ and refined with full-matrix least-squares on *F* ^2^ ^40^ using Olex2 as a graphical interface.^41^ The non-hydrogen atoms were refined anisotropically. Hydrogen atoms were located in difference Fourier maps and included as fixed contributions riding on attached atoms with isotropic thermal displacement parameters 1.2 or 1.5 times those of the respective atom. Mercury^42^ was used for the analysis and visualization of the structure and also for graphic material preparation. The CIF file is deposited in the Cambridge Structural Database (CSD) under the CCDC number 2427693. Copies of the data can be obtained free of charge at https://www.ccdc.cam.ac.uk/structures/.

#### 2.4.2 Fourier-Transformed Infrared

Fourier-transform infrared (FT-IR) spectroscopic measurements were conducted on a Hyperion 3000 (Bruker, Massachusetts, USA) instrument equipped with a single-reflection diamond crystal platinum ATR unit and OPUS data collection program. The scanning range was from 4000 to 400 cm^–1^ with a resolution of 4 cm^–1^.

#### 2.4.3 Differential Scanning Calorimetry

Differential scanning calorimetry (DSC) studies were carried out with a NETZSCH STA 449F5 calorimeter (NETZSCH Group, Germany). Experimental conditions: alumina (Al_2_O_3_) crucibles of 85 μL volume, an atmosphere of dry nitrogen with 250 mL/min flow rate, and heating rates of 5 °C/min with the non-isothermal method from 25 to 250 °C. To calibrate the calorimeter, indium of 99.99% purity was used (mp 156.4 °C; DH: 28.14 J/g).

#### 2.4.4 Thermodynamic Stability Analysis

The thermodynamic stability of the materials was evaluated under accelerated aging conditions. 200 mg of MTF–DKT, DKT, and MTF were placed in watch glasses and left at 40 °C and 75% RH in a Memmert HPP110 climate chamber (Memmert, Schwabach, Germany). The integrity of the solid forms under the conditions mentioned above was periodically monitored using PXRD for 4 months.

Additionally, the stability in an aqueous suspension of the MTF–DKT salt was evaluated through slurry experiments in which an excess of powder sample was added to 0.5 mL of (1) buffer KCl 0.01 M at a pH of 1.2 and (2) buffer PBS 0.01 M at a pH of 6.8. After 24 h of stirring at 25 °C in sealed vials, the solids were collected, filtered, dried, and analyzed with PXRD to evaluate the stability and crystallinity of the salt.

#### 2.4.5 Solubility Studies

The solubility studies were undertaken following the shake-flask method^43^ using buffer KCl pH 1.2 and buffer PBS pH 6.8, to simulate the physiological conditions of the stomach and intestine, respectively, and to assess the behavior of the drugs in the gastrointestinal environment. In these experiments, saturated solutions of MTF–DKT and DKT were prepared by adding an excess of solid to 10 mL of each buffer and stirring for 24 h at 25 °C until thermodynamic equilibrium was reached. During this period, aliquots of the solution were filtered through 0.22 μm syringe filters and diluted to achieve a measurable concentration. Samples were evaluated and measured at 260 nm, the DKT maximum of absorbance (Figure S2), using a UV–vis Varian Cary 50 (Agilent Technologies, Santa Clara, CA, USA).

#### 2.4.6 Electron Microscopy

Scanning electron microscopy (SEM) was performed using a JEOL SEM microscope, model JSM 6490-LV (JEOL Inc.), with a tungsten filament operating at 10 kV. Before analysis, the samples were placed in a JEOL EMDSC-U10A vacuum desiccator (JEOL Inc., MA, USA).

## 3 Results and Discussion

### 3.1 Salt Synthesis

The mechanochemical synthesis of the novel MPM was performed by LAG due to its efficiency, reproducibility, and minimal organic solvent requirement compared to conventional synthesis methods.^44^ In this work, the formation of a new crystalline material was evidenced by PXRD and FT-IR. Figure 2 shows (a) the PXRD diffractograms and (b) the FT-IR spectra of DKT, MTF, and the product obtained from an LAG reaction of a 1:1 stoichiometric mixture of MTF and DKT, using ethanol as a liquid additive. In Figure 2a, the product of the LAG exhibits a different PXRD pattern when compared with the reaction components, indicating the formation of a new crystalline material. The absence of reflections attributed to MTF and DKT also suggests the correct stoichiometry of the reaction and complete conversion of the components into this new phase. As a complementary technique, FT-IR can provide valuable information about the functional groups involved in the formation of the new material, enabling the distinction between the formation of cocrystals or salts. In Figure 2b, the DKT spectrum exhibits a sharp and intense band at 1727 cm^–1^ corresponding to the asymmetric stretching mode of the carboxylic acid (C=O) group, in addition to a signal at 1652 cm^–1^ associated with the stretching mode of the C=O (ketone) group. In the 3200 cm^–1^ region, a broad band is observed, which is attributed to the stretching mode of the —OH group. After the reaction with MTF, the (C=O) stretching band of the carboxylic acid group completely shifts to lower wavenumbers, indicating the ionization of the group and the possible formation of a molecular salt. Furthermore, the signals of the C=O and —OH groups shift to 1653 and 3158 cm^–1^, respectively, indicating changes in the vibration modes due to interactions with other functional groups. In the MTF spectrum, a series of bands between 3420 and 3090 cm^–1^ are observed, attributed to the asymmetric and symmetric stretching of the —NH_2_ group and the stretching mode of the —NH groups. Additionally, a less defined band at 1537 cm^–1^ is associated with the bending modes of the —NH groups. All these signals shift after the reaction with DKT, which indicates the interaction of these functional groups. Indeed, in the MTF–DKT spectrum, a characteristic double band at 1574 and 1557 cm^–1^ is observed, corresponding with such a bending mode of the —NH groups. This double band is not present in MTF, making it distinctive of this new phase, and it can be used as a reference for charge studies in nAp.

**Figure 2:**
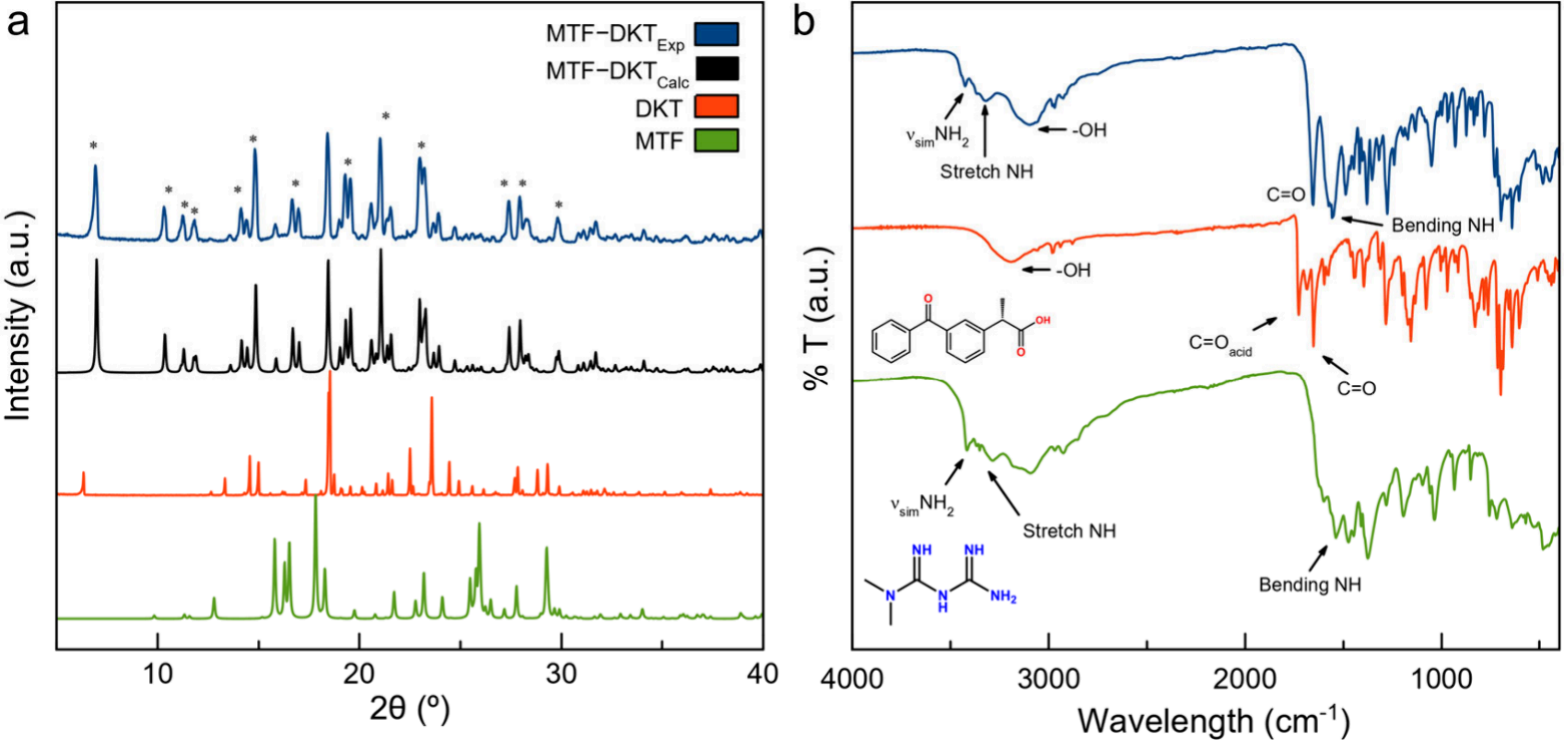
(a) PXRD diffractograms and (b) FT-IR spectra of MTF, DKT, the product of the LAG reaction, and the simulated diffractogram based on the MTF–DKT crystal structure.

Once the obtention of a novel phase was confirmed, the slow evaporation of saturated solutions of the product of LAG in ethanol allowed us to obtain suitable crystals for further crystal structure studies by SCXRD. Additionally, the simulated powder pattern derived from this structure was used to confirm the purity, crystallinity, and reproducibility of the phase in the synthesis reactions.

### 3.2 Crystal Structure Analysis

The crystal structure of the novel phase determined by SCXRD corroborated the PXRD and FT-IR results and confirmed the transfer of a proton from the carboxylic acid group of DKT to the most basic nitrogen group of MTF, resulting in the formation of the novel molecular salt MTF–DKT. The experimental electron density map is in agreement with these findings, which are further confirmed by the analysis of the C–O bond distances in the carboxylate group of the NSAID, showing Δ*D*_C–O_ values similar to those observed in salts, ranging from 0.008 to 0.024 Å.^45,46^ The crystallographic data are summarized in Table 1, while the information about hydrogen bonding is provided in Table S1.

**Table 1:** Crystallographic Data and Refinement Details for MTF–DKT

**Compound name**	**MTF–DKT**
Formula	C_40_H_50_N_10_O_6_
Molecular weight	766.90
Crystal system	Monoclinic
Space group	*P*2_1_(4)
*a*/Å	14.059(3)
*b*/Å	9.178(2)
*c*/Å	16.920(4)
α/°	90
β/°	112.416(7)
γ/°	90
*V*/Å^3^	2018.3(8)
*Z*	2
Dc/g cm^–3^	1.262
*F*(000)	816
Reflections collected	39277
Unique reflections	7068 [*R*(int) = 0.0779]
Data/restraints/parameters	7068/1/531
Goodness-of-fit on *F* ^2^	1.002
*R*_1_ (*I* > 2σ(*I*))	0.0483
w*R*_2_ (*I* > 2σ(*I*))	0.1111

MTF–DKT crystallizes in the monoclinic P2_1_ space group, with two MTF^+^ cations (MTF1, MTF2) and two DKT^–^ anions (DKT1, DKT2) in the asymmetric unit, which are chemically equivalent but crystallographically independent. First, the dihedral angle defined by the guanidinium group and the dimethylamine group differs between the two MTF molecules: in MTF1, this angle is 56.32°, while, in MTF2, it is 55.57°. Second, only the DKT1 molecule exhibits disorder in the carboxylate group, which is positioned in two alternate locations. Figure 3a shows the asymmetric unit of this molecular salt, illustrating the disordered DKT anion with the major contributing component (66%). MTF^+^ molecules interact with DKT^–^ through a discrete electrostatic hydrogen bond involving the guanidinium···carboxylate group, forming the heterosynthon *D*_1_^1^(2). The MTF–DKT pairs are connected through hydrogen bonds *R*_2_^2^(8) between the amine groups of the MTF^+^ cations, which are established around an inversion center. Additionally, asymmetric units are associated through hydrogen interactions *R*_2_^2^(8) involving the carboxyl group of DKT^–^ and the −NH group of MTF^+^, resulting in 1D undulated chains that extend along the *b*-axis. In these chains, DKT^–^ molecules are positioned at the exterior, while the MTF^+^ molecules are shielded inside (Figure 3b). These crystallographic features will have important implications for the physicochemical properties of the salt. Finally, chains associate through CH···π interactions to form 2D layers that extend parallel to the (1, 0, –1) plane of the crystal (Figure 3c) and these layers stack to form the 3D supramolecular structure, maintained by weak non-covalent hydrophobic interactions.

**Figure 3:**
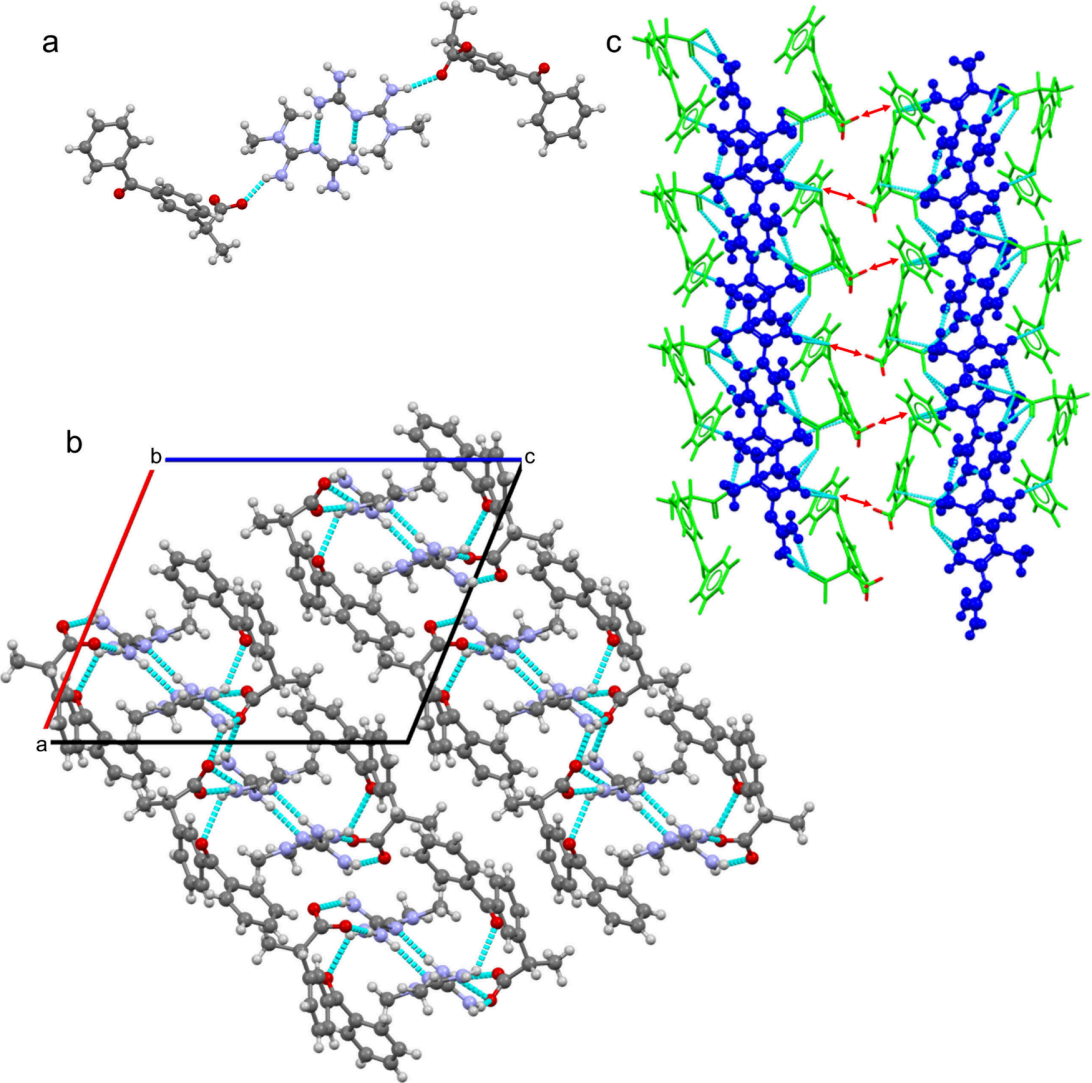
(a) Asymmetric unit of MTF–DKT. (b) 1D undulated chain structure observed in MTF–DKT along the *b* axis. (c) Formation of 2D layers through CH···π interactions between adjacent chains. MTF^+^ is shown in blue and DKT^–^ in green.

### 3.3 Study of MTF–DKT Properties

#### 3.3.1 Thermal Stability

DSC analysis was performed to evaluate the impact of salt formation on the thermal stability and determine the melting point of MTF–DKT. Figure 4a shows the DSC traces of the salt and the parent APIs in which the endothermic events correspond to the melting point. The presence of a single and well-defined endothermic peak confirms the stability of the salt below the melting point. No transformations or dissociation phenomena occur, supporting the phase purity already observed by PXRD. In this case, MTF–DKT presents a melting point of 152.9 °C, indicating a significant improvement in the thermal stability compared to that of both parent APIs, nearly doubling the melting point of DKT (75 °C). These results differ from the typical behavior of salts and cocrystals in which the melting point of the novel material usually falls in between the melting point of the components.^47–49^

**Figure 4:**
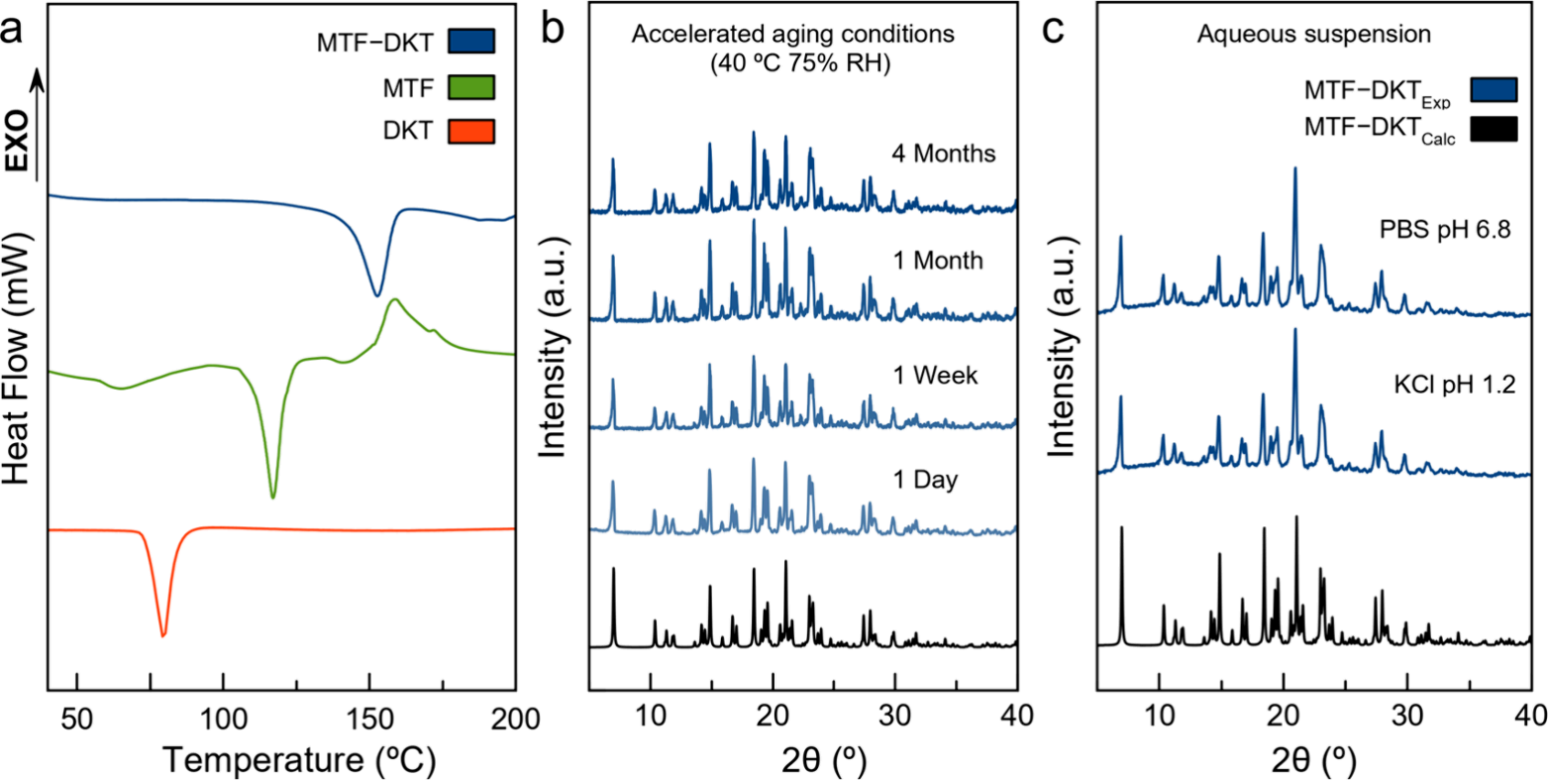
(a) DSC curves of DKT, MTF, and MTF–DKT. (b) PXRD diffractograms of MTF–DKT under accelerated aging conditions and (c) in aqueous suspension (buffer KCl pH 1.2 and PBS pH 6.8) after 24 h.

#### 3.3.2 Thermodynamic Stability

Thereafter, the thermodynamic stability of MTF–DKT was evaluated under accelerated aging conditions (40 °C and 75% RH) for 4 months to dismiss processes such as dissociation, hydration, or polymorphic transitions. Figure 4b shows the PXRD patterns of the salt under these conditions. The overall stability of the new salt is demonstrated by the absence of changes in the PXRD diffractogram or changes in intensity (indicative of changes in crystallinity). As expected, salt formation with DKT provides MTF with excellent protection against humidity. Note that the MTF-free form could not even be evaluated due to its low stability and high hygroscopicity, which resulted in liquefaction within the first few hours of exposure to humidity (Figure S3). The thermodynamic stability in aqueous suspensions of MTF–DKT was assessed through slurry experiments in buffer KCl (pH 1.2) and PBS (pH 6.8) at 25 °C. Figure 4c shows the PXRD diffractograms for the solids, which were filtered and air-dried after 24 h of stirring. In any case, visible changes in color or texture were observed. No changes were detected in the diffractograms that could be associated with phase transitions or hydration phenomena, indicating that the new molecular salt is stable in an aqueous solution under the pH and temperatures tested during at least 24 h, which is larger than the expected time of a LADME process for any oral drug.

The overall improvement of stability over MTF is attributed to the molecular arrangement in the crystal structure, as MTF–DKT forms infinite chains in which DKT molecules are exposed to the exterior while MTF molecules are covered in the interior. This arrangement allows the DKT molecules to act as a protective shield, safeguarding the MTF from the attack of water molecules and other agents in the medium. These results are comparable to those obtained by Sun et al.,^50^ who demonstrated that epalrestat–metformin cocrystals exhibited superior resistance to humidity compared to the parent APIs, due to their molecular arrangements that prevent hydration-induced degradation.

In addition to providing significant resistance to humidity, the novel crystal structure and the intricate hydrogen bonding network that connects the MTF and DKT molecules enhance the thermal stability of both APIs, as shown in the DSC. Similar results have already been reported, demonstrating the great potential of this type of pharmaceutical solids.^25,51,52^

#### 3.3.3 Solubility and Dissolution Profile of MTF–DKT

Considering the aim of this work, the solubility studies focus on the quantification of DKT, as MTF exhibits excellent solubility and does not pose a limiting factor for nAp loading. Thereby, the thermodynamic solubility and dissolution profile of DKT and MTF–DKT were evaluated under physiological conditions relevant to drug absorption and metabolism (aqueous buffer solution, KCl pH 1.2 and PBS pH 6.8). The solubility values of DKT and MTF–DKT measured at 260 nm during 24 h are gathered together in Table S2.

The thermodynamic solubility in PBS of DKT reached by the MTF–DKT salt is 456 mg/mL, which represents a 2150-fold increase compared with DKT alone (0.21 mg/mL). In KCl solution at pH 1.2, solubility values of 0.1 mg/mL were obtained for DKT alone, while the MTF–DKT salt reached concentrations of 161 mg/mL, increasing the solubility at acidic pH 1600-fold (Figure 5). It is noteworthy that the dissolution profile shape is similar for both DKT and MTF–DKT, as they both exhibit a spring-parachute behavior, with supersaturation occurring within the first few minutes. In the salt, this phenomenon allows peak DKT concentrations of 567 and 168 mg/mL at pH 6.8 and 1.2, respectively. These results demonstrate exceptional solubility for the salt, greatly increasing the concentration of DKT in solution.

**Figure 5:**
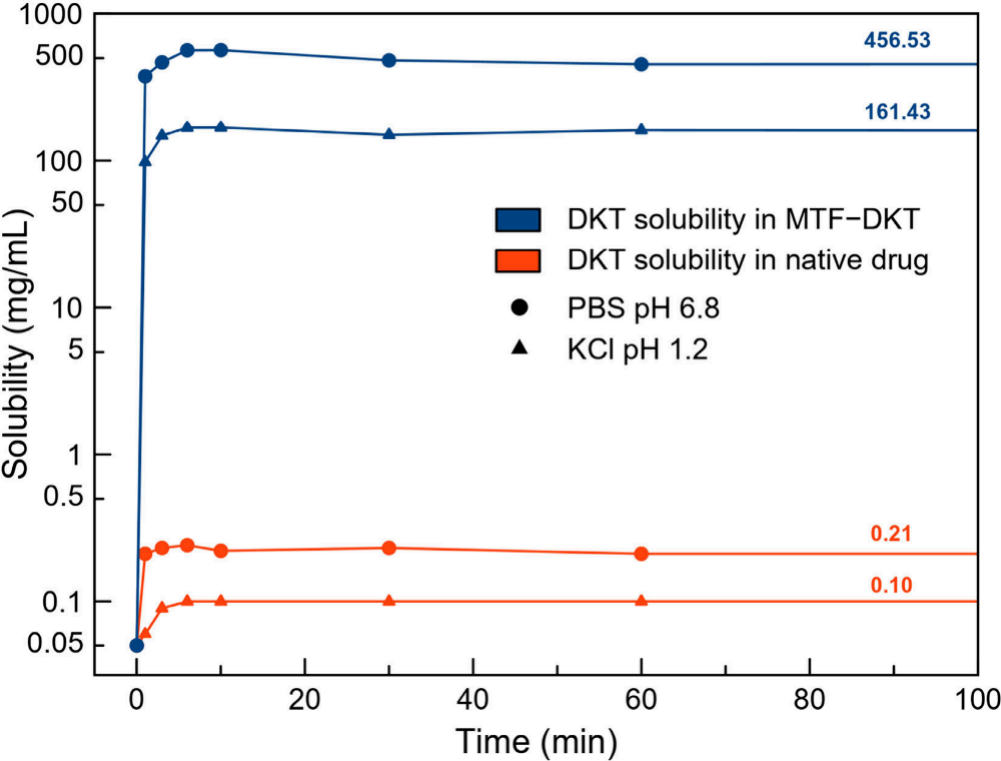
Solubility profile of DKT (red) and MTF–DKT (blue) in KCl pH 1.2 (▲) and PBS pH 6.8 (●).

The significant solubility improvement observed for DKT in the new molecular salt is also a consequence of the arrangement of the APIs in the supramolecular structure. Even if the MTF molecules are located inside of the undulated chains, their higher solubility facilitates the disintegration of such chains and the release of DKT molecules into the medium. Moreover, the presence of MTF within the chains in the new salt arrangement disrupts the strong DKT dimers present in the native structure, enhancing the solubility.

The reported thermodynamic stability in the previous section also allows for the estimation of MTF solubility in these tests, considering both the molecular weight of MTF and the 1:1 stoichiometry within the salt. In acidic KCl solution, MTF reaches an equilibrium concentration of 54 mg/mL, while in PBS media equilibrium concentrations of 150 mg/mL are reached, with a peak concentration of 191 mg/mL. These values are comparable to the solubility of MTF·HCl under physiological conditions; however, in MTF–DKT, we are able to substitute the ion Cl^–^ with an API that has intrinsic therapeutic activity.

### 3.4 Drug Loading Assays

The most common route for obtaining doped nAp is based on the *in situ* incorporation of the dopant agent during the nanoparticle synthesis in a one-pot assay.^53,54^ However, the incorporation of DKT and, primarily, MTF–DKT presents more complexity due to several considerations.

The nAp used in this study is obtained through thermal decomplexation of Ca^2+^/citrate/phosphate/carbonate solutions at 80 °C. Citrate incorporation into nAp provides a biomimetic character that enhances the biological properties of the material and adds surface functional groups that facilitate the adsorption of other molecules during doping. However, the presence of citrate in the reaction medium also competes with DKT in forming a molecular salt with MTF.^27^ Furthermore, the strong basic nature of MTF makes this molecule highly reactive and suitable for salt formation with either organic or inorganic counterions.^55–58^ Finally, the high temperatures used during nAp synthesis (80 °C) are detrimental to thermolabile organic molecules such as MTF, which has been shown to be very unstable under high humidity and temperature conditions. In this context, the incorporation of MTF and DKT, either as free agents or in the MTF–DKT phase, is not viable for one-pot doping of nAp, as it would severely affect the formation and stability of the MTF–DKT salt, the properties of the synthesized nAp, and their loading efficiency.

An alternative methodology to avoid these problems and maximize DKT loading is proposed by Carmona et al.^37^ These authors suggest postsynthesis functionalization of nanoparticles via chemical precipitation. This methodology not only prevents altering the properties of the nanoparticles but also avoids the loss of doping efficiency due to washing cycles. In this way, when the dopant agent is provided with sufficient aqueous solubility to reach the required concentration, maximum loading efficiency is ensured as the entire amount of dopant agent remains bound to the nanoparticle surface or occluded within the material.

The excellent solubility achieved by MTF–DKT allows us to employ this methodology for loading of the nAp. In this assay, the first step is to conduct a screening to identify the maximum amount of dopant agent that can be incorporated into the nAp. To establish a comparison, this methodology will be carried out using DKT and MTF–DKT. For this purpose, 50 mg of nAp was mixed with 1 mL of solutions with increasing concentrations of DKT and MTF–DKT. After 4 h of stirring, the suspension was lyophilized and analyzed by PXRD. Figure 6a shows the PXRD diffractograms of nAp–MTF–DKT obtained at concentrations ranging from 1 to 50 mg/mL. The coprecipitation of nAp with the dopant agent in the crystalline phase indicates that the loading limit in nAp has been exceeded, resulting in a mixture of doped nAp phases and excess dopant.

**Figure 6:**
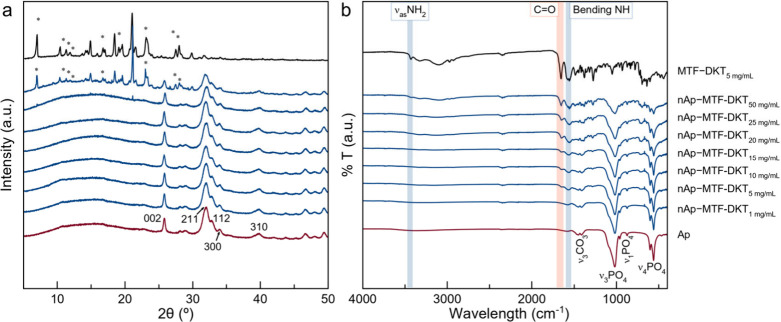
(a) PXRD diffractograms and (b) FT-IR spectra of nAp–MTF–DKT samples.

At concentrations below 25 mg/mL MTF–DKT, the diffractograms show the fundamental reflections of Ap (ASTM card file no. 9-432): 2θ = 25.87° (plane (002)), 31.77° (211), 32.19° (112), 32.90° (300), 33.9° (202), and 39.81° (310). Other minor reflections associated with the Ap phase are also observed within the 2θ range of 40° to 45°. Additionally, the broad reflections in the diffractograms indicate the nanoscale dimensions of the crystalline domains.^59,60^ It was also observed that no additional calcium phosphate phases were detected, such as octacalcium phosphate (OCP, ASTM card no. 26-1056) or brushite (DCPD, ASTM card no. 9-77). This is confirmed by the absence of reflections at 2θ of 4.8° and 11.6°, corresponding to the (100) plane of OCP and the (020) plane of DCPD. These data ensure that the chemical precipitation of MTF–DKT via lyophilization does not affect the crystallinity or nanometric size of the nAp.

At a 25 mg/mL concentration of MTF–DKT, weak signals corresponding to MTF–DKT appear. These signals become much more evident when the concentration is increased to 50 mg/mL as a result of a massive precipitation of the salt in the crystalline phase. In parallel and to discard the amorphous precipitation of MTF–DKT, the same protocol was performed using MTF–DKT solutions in the absence of nAp. The powder pattern from the lyophilization of a 5 mg/mL MTF–DKT solution is shown in Figure 6a as a reference. These results indicate that the maximum concentration of MTF–DKT accepted by the nAp is 20 mg/mL, representing 28.57% by weight relative to the total amount of precipitate (nAp + dopant). We should bear in mind that the dopant agent is a drug–drug salt; thus, in order to compare the amount of DKT loaded in the MTF–DKT salt with the reference API, it should be considered that DKT represents 66.3% by weight in MTF–DKT. With these considerations, the maximum concentration of DKT (in salt phase) incorporated into the nAp is 13.26 mg/mL, which corresponds to 18.95% w/w. Notably, the concentration of MTF–DKT needed to saturate the nAp is only 3.3% of the total solubility of the salt (approximately 600 mg/mL in PBS pH 6.8). In contrast, the limited aqueous solubility of DKT only allows for a maximum concentration of DKT (alone), incorporated into the nAp, of 0.2 mg/mL, representing 0.4% of the total weight of nAp–DKT, which is 66 times lower when compared to MTF–DKT. As expected, at these concentrations, crystalline precipitation of DKT does not occur since the nAp saturation has not been reached. These results are not shown in Figure 6, as all diffractograms associated with DKT native drug loading correspond to the Ap phase (Figure S4). All concentration values used in these assays, as well as their respective weight percentages, are listed in Table 2.

**Table 2:** Concentration of DKT and MTF–DKT (mg) in 1 mL of an nAp Suspension (50 mg)

**DKT**	**% w/w**	**MTF–DKT**	**% w/w**	**DKT in** MTF–DKT	**% w/w**
0.1	0.20	1	1.96	0.66	1.30
0.2	0.40	5	9.09	3.32	6.03
		10	16.67	6.63	11.05
		15	23.08	9.95	15.30
		20	28.57	13.26	18.95
		25	33.33	16.58	22.10
		50	50.00	33.16	33.16

Once the loading limit of the nAp has been established, the presence of the dopant agent must be confirmed, because the dissociation of the molecular salt might occur, thereby multiplying the possible scenarios with which the nAp can be doped, i.e., (1) only DKT, (2) only MTF, (3) DKT and MTF as free molecules, and (4) the MTF–DKT salt as a whole, maintaining non-covalent interactions. In this context, FT-IR allows for the study of interactions between functional groups, confirming the adsorption of discrete molecules or the salt. Figure 6b shows the FT-IR spectra of nAp loaded with different concentrations of MTF–DKT.

As expected, the low DKT loading percentage in nAP–DKT makes its detection by FT-IR impossible, in line with the previous results of PXRD. At all concentrations, only the characteristic signals of Ap are observed in the 400–1800 cm^–1^ region, including the asymmetric stretching of the PO_4_^3–^ groups (υ_3_PO_4_) in the 1000–1100 cm^–1^ region, the symmetric stretching υ_1_PO_4_ at 958–960 cm^–1^, and the bending modes υ_4_PO_4_ at 608 and 1564 cm^–1^ and υ_2_PO_4_ at 470 cm^–1^. The presence of carbonate groups (CO_3_^2–^) is confirmed by bands at 1414 and 1473 cm^–1^ (υ_3_CO_3_) and an intense band at 873 cm^–1^ (υ_2_CO_3_), which is indicative of the biomimetic character of the nAp. In the case of MTF–DKT loading, in addition to the above referenced Ap signals, the characteristic signals of the salt are observed, including the double band at 1574 and 1557 cm^–1^ attributed to the bending mode of —NH groups and a band at 1653 cm^–1^ corresponding to the C=O stretching mode (ketone). These signals are detectable from a concentration of 5 mg/mL and increase in intensity as the incorporated salt concentration rises. When nAp saturation (>25 mg/mL) is reached, the intensity of nAp signals decreases relative to the salt signals due to the phase mixture. The absence of a well-defined band at 1727 cm^–1^ corresponding to the asymmetric stretching mode of the carboxylic acid C=O group of DKT rules out the incorporation of free DKT molecules. This evidence supports the notion that MTF–DKT molecules are adsorbed on the surface or inside the aggregated nAp.

The incorporation of the salt as a whole explains the high DKT loading capacity compared to the native API. It has been demonstrated that the presence of negatively charged ionic groups endows nAp with a strong affinity for nitrogen-containing molecules such as MTF.^61–63^ This affinity for nAp, along with the tendency to form salts with DKT, allows MTF to act as a bridging molecule between nAp and DKT, enabling a very high DKT loading capacity. This is an additional advantage of using MPM to increase the loading limit since it also allows the incorporation of another therapeutic agent like MTF.

Lastly, to evaluate morphological changes in the nAp that might be associated with the chemical precipitation process, a detailed analysis was performed by using SEM. Figure 7 shows the images taken for nAp and nAp–MTF–DKT samples obtained with concentrations of 10, 20, and 50 mg/mL, as well as for the crystalline precipitate of a 5 mg/mL MTF–DKT solution. The images show nAp with a homogeneous morphology typical of biomimetic nAp obtained by thermal decomplexation with a particle size below 50 nm.^64,65^ However, due to the long maturation times, aggregation and agglomeration phenomena prevail, resulting in the crystals forming into spherical aggregates.^65^ Functionalization of nAp with MTF–DKT does not produce observable changes in terms of the nAp morphology and size. When the concentration is increased beyond the loading capacity, large crystals of the salt are observed along with the nAp.

**Figure 7:**
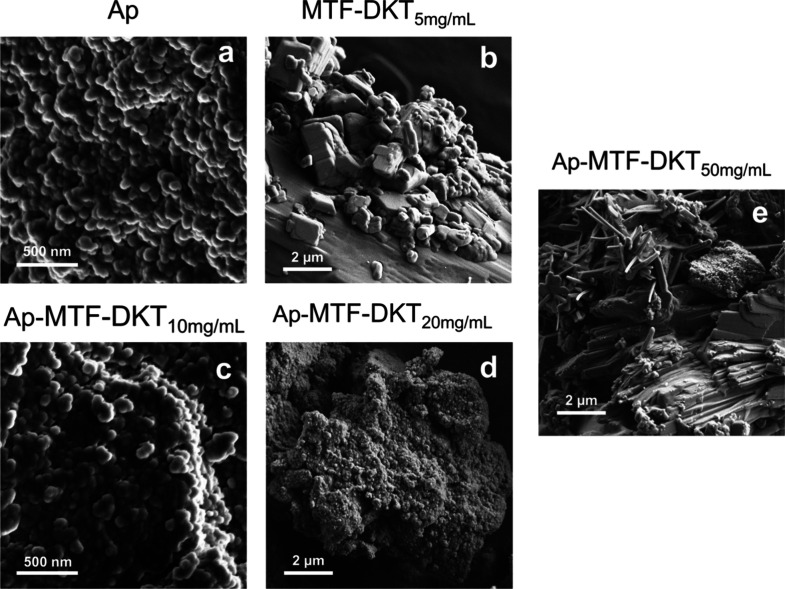
SEM images of (a) nAp, (b) MTF–DKT_5mg/mL_, (c) nAp–MTF–DKT_10mg/mL_, (d) nAp–MTF–DKT_20mg/mL_, and (e) nAp–MTF–DKT_50mg/mL_.

MTF–DKT crystals grown in the presence of nAp exhibit a smaller size and an elongated morphology, forming prismatic needles, which differ from the crystals precipitated in the absence of nAp, which are characterized by larger particle size and a block-like prismatic morphology. These findings suggest that MTF–DKT crystal nucleation in the presence of nAp may initiate from the nAp surface, thereby creating a greater number of nucleation sites and resulting in smaller crystals. These results not only corroborate the deposition of the salt on the nAp but also demonstrate that the loading process is harmless to the biomimetic properties of nAp obtained by thermal decomplexation.

## 4 Conclusions

This study demonstrates the potential of the presented strategy to significantly enhance the drug-loading capacity of BCS II and IV drugs in apatite nanoparticles, addressing the solubility limitations that commonly restrict their use in oral drug delivery systems with the appropriate design of MPMs. The crystal structure arrangements achieved in the novel MTF–DKT molecular salt led to an extraordinary solubility enhancement that resulted in a remarkable 50-fold increase in loading capacity compared with native DKT, shifting the limiting factor from DKT solubility to the surface area of the nAp. While this approach was validated using nAp, commonly associated with bone-related applications, the versatility shown herein could be extended to a broad range of materials with diverse applications and greater capacities for dopant incorporation, including other types of nanoparticles, surfaces, polymeric scaffolds, and liposomes. Even with the promising results of this work, further in vitro and in vivo studies will be required to assess the biocompatibility, long-term stability, and potential toxicity of the hybrid system here reported. Furthermore, scalability challenges should be explored to facilitate the transition from laboratory-scale synthesis to industrial manufacturing. Addressing these limitations will provide a more comprehensive understanding of the potential of this novel drug delivery strategy.

## Supplementary Material



## Notes

Supplementary tables and figures showing (1) PXRD patterns of MTF·HCl and the MTF-free form; (2) UV–vis spectra of DKT and MTF; (3) photograph of MTF after 4 h under accelerated aging conditions; (4) PXRD diffractograms and FT-IR spectra of nAp–DKT samples; (5) hydrogen bonds present in MTF–DKT; (6) achieved concentration of DKT (mg/mL) using the native form of the drug and the MTF–DKT salt, measured in buffer KCl pH 1.2 and PBS pH 6.8 (PDF)
