# Supplementary material for: Enhanced Drug Loading Capacity Using the Dual Metformine–Dexketoprofren Salt on Nanoapatite Materials
Source: Mol Pharm. 2025 Apr 25;22(6):3377–87. doi: 10.1021/acs.molpharmaceut.5c00264 (PMC12135057; doi:10.1021/acs.molpharmaceut.5c00264)
Supplement: Supplementary file 1 [file mp5c00264_si_001.pdf]

Supplementary Material

Enhanced drug loading capacity using the dual Metformine-Dexketoprofren salt on nanoapatite materials

Francisco J. Acebedo-Martínez,[1] Alicia Domínguez-Martín[2], Carolina Alarcón-Payer[3], Cristóbal Verdugo-Escamilla,[1] Jaime Gómez-Morales,[1,], Duane Choquesillo-Lazarte[1,*]*

[1] Laboratorio de Estudios Cristalográficos, IACT, CSIC-Universidad de Granada, Avda. de las Palmeras 4, 18100 Armilla, Spain.

[2] Department of Inorganic Chemistry, Faculty of Pharmacy, University of Granada, 18071 Granada, Spain.

[3] Servicio de Farmacia, Hospital Universitario Virgen de las Nieves, 18014 Granada, Spain.

Figure S1. PXRD patterns of MTF·HCl and MTF free form.
Figure S2. UV-Vis spectra of DKT and MTF.
Figure S3. MTF after 4 hours under accelerated ageing conditions.
Figure S4. (a) PXRD diffractograms and (b) FT-IR spectra of nAp-DKT samples.

Table S1. Hydrogen bonds present in MTF–DKT.
Table S2. Achieved concentration of DKT (mg/mL) using the native form of the drug and the MTF–DKT salt, measured in buffer KCl pH 1.2 and PBS pH 6.8.

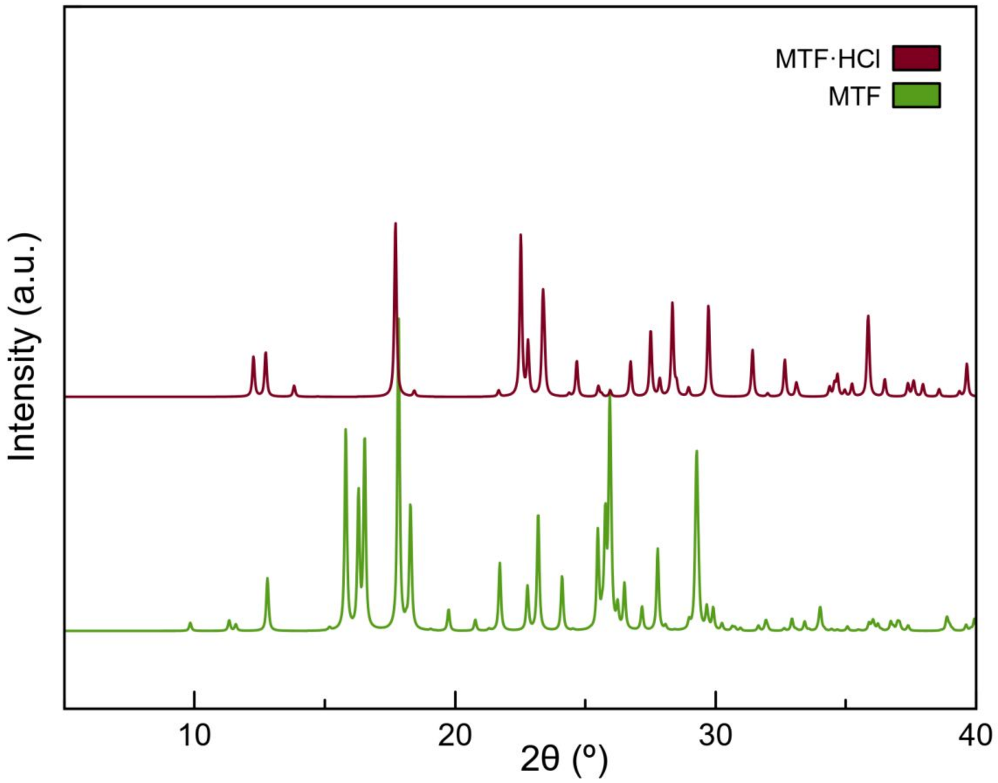

Figure S1. PXRD patterns of MTF·HCl and MTF free form.

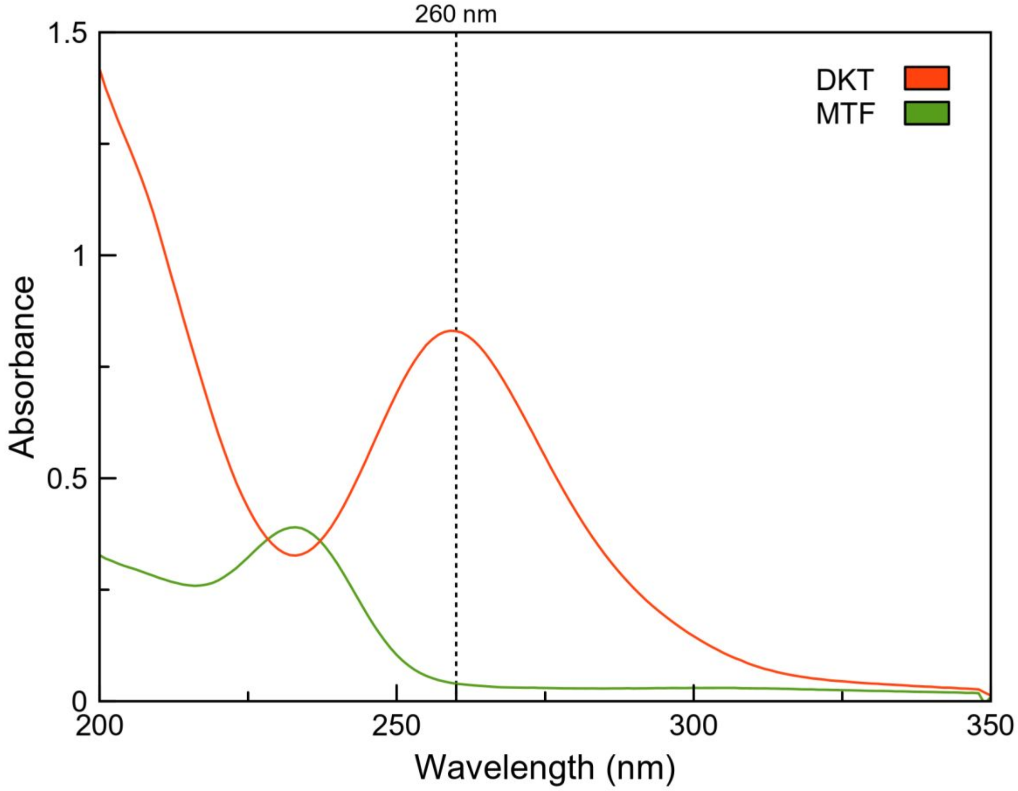

Figure S2. UV-Vis spectra of DKT and MTF.

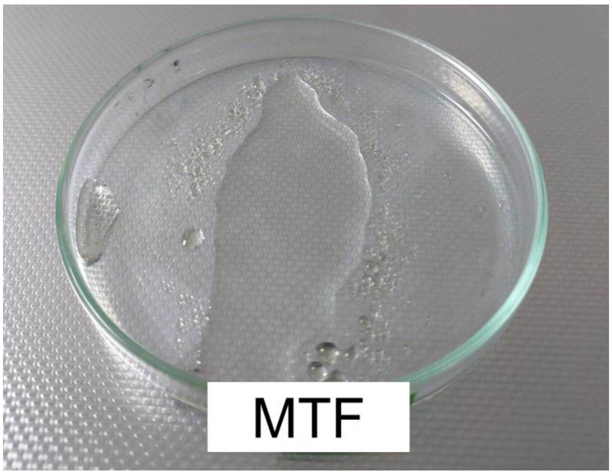

Figure S3. MTF after 4 hours under accelerated ageing conditions.

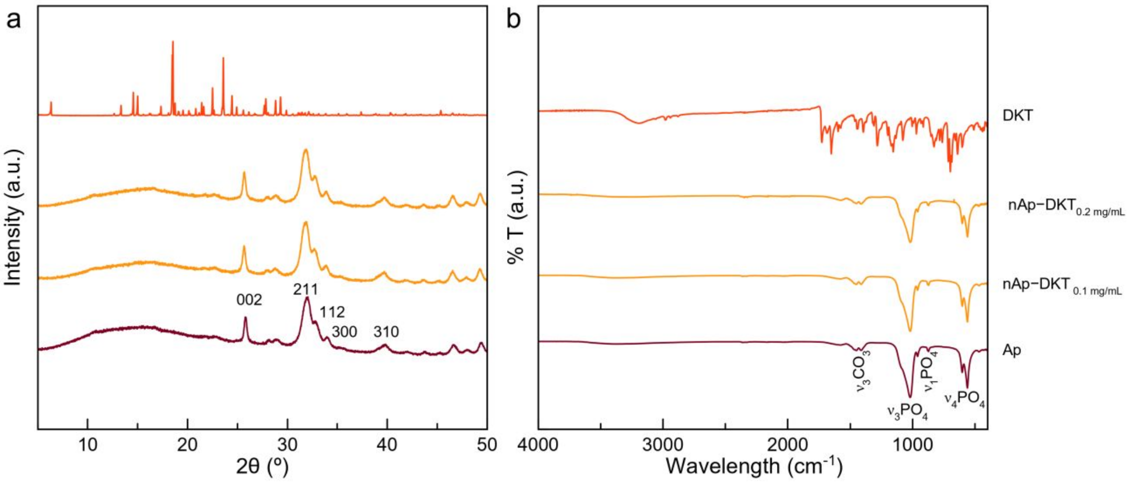

Figure S4. (a) PXRD diffractograms and (b) FT-IR spectra of nAp-DKT samples.

Table S1. Hydrogen bonds present in MTF–DKT.

MTF–DKT	D-H···A	d(D-H)	d(H···A)	d(D···A)	<(DHA)
	N(20)-H(20A)···O(5A)[#1]	0.86	2.08	2.857(17)	150.4
	N(20)-H(20A)···O(5B)[#1]	0.86	2.09	2.92(4)	163.9
	N(20)-H(20B)···O(5A)[#2]	0.86	2.16	2.865(14)	139.2
	N(20)-H(20B)···O(5B)[#2]	0.86	2.40	3.05(5)	133.0
	N(22)-H(22A)···O(5A)[#1]	0.86	2.15	2.908(17)	147.3
	N(22)-H(22B)···N(51)	0.86	2.15	3.003(6)	173.8
	N(25)-H(25A)···O(4A)	0.86	1.92	2.642(9)	140.3
	N(25)-H(25A)···O(4B)	0.86	2.06	2.89(3)	161.5
	N(25)-H(25B)···O(13)[#1]	0.86	2.44	3.029(6)	126.1
	C(55)-H(55A)···O(32)	0.96	2.45	3.160(6)	130.6
	N(1)-H(1D)···O(32)	0.86	2.05	2.892(6)	164.8
	N(1)-H(1E)···O(41)[#3]	0.86	2.42	3.100(5)	137.0
	N(49)-H(49A)···N(23)	0.86	2.18	3.038(6)	173.4
	N(49)-H(49B)···O(32)[#3]	0.86	2.11	2.959(5)	169.6
	N(50)-H(50A)···O(33)[#3]	0.86	2.11	2.946(6)	162.8
	Symmetry operation:				
	#1 x,y+1,z #2 -x+2,y+1/2,-z+1 #3 x,y-1,z				

Table S2. Achieved concentration of DKT (mg/mL) using the native form of the drug and the MTF–DKT salt, measured in buffer KCl pH 1.2 and PBS pH 6.8.

(mg/mL)	DKT		DKT in MTF-DKT		
Time (min)	pH 1.2	pH 6.8	Time (min)	pH 1.2	pH 6.8
1	0.06	0.21	1	98.15	377.61
3	0.09	0.23	3	148.36	469.13
6	0.10	0.24	6	168.33	566.84
10	0.10	0.22	10	168.62	567.80
30	0.10	0.23	30	150.32	483.90
60	0.10	0.21	60	161.87	455.30
120	0.10	0.21	120	160.97	456.07
1440	0.10	0.21	1440	161.43	456.43
